# Investigating Bacterial Sources of Toxicity as an Environmental Contributor to Dopaminergic Neurodegeneration

**DOI:** 10.1371/journal.pone.0007227

**Published:** 2009-10-06

**Authors:** Kim A. Caldwell, Michelle L. Tucci, Jafa Armagost, Tyler W. Hodges, Jue Chen, Shermeen B. Memon, Jeana E. Blalock, Susan M. DeLeon, Robert H. Findlay, Qingmin Ruan, Philip J. Webber, David G. Standaert, Julie B. Olson, Guy A. Caldwell

**Affiliations:** 1 Department of Biological Sciences, The University of Alabama, Tuscaloosa, Alabama, United States of America; 2 Department of Neurology, Center for Neurodegeneration and Experimental Therapeutics, University of Alabama at Birmingham, Birmingham, Alabama, United States of America; 3 Department of Neurobiology, University of Alabama at Birmingham, Birmingham, Alabama, United States of America; National Institutes of Health, United States of America

## Abstract

Parkinson disease (PD) involves progressive neurodegeneration, including loss of dopamine (DA) neurons from the *substantia nigra*. Select genes associated with rare familial forms of PD function in cellular pathways, such as the ubiquitin-proteasome system (UPS), involved in protein degradation. The misfolding and accumulation of proteins, such as α-synuclein, into inclusions termed Lewy Bodies represents a clinical hallmark of PD. Given the predominance of sporadic PD among patient populations, environmental toxins may induce the disease, although their nature is largely unknown. Thus, an unmet challenge surrounds the discovery of causal or contributory neurotoxic factors that could account for the prevalence of sporadic PD. Bacteria within the order *Actinomycetales* are renowned for their robust production of secondary metabolites and might represent unidentified sources of environmental exposures. Among these, the aerobic genera, *Streptomyces*, produce natural proteasome inhibitors that block protein degradation and may potentially damage DA neurons. Here we demonstrate that a metabolite produced by a common soil bacterium, *S. venezuelae*, caused DA neurodegeneration in the nematode, *Caenorhabditis elegans*, which increased as animals aged. This metabolite, which disrupts UPS function, caused gradual degeneration of all neuronal classes examined, however DA neurons were particularly vulnerable to exposure. The presence of DA exacerbated toxicity because neurodegeneration was attenuated in mutant nematodes depleted for tyrosine hydroxylase (TH), the rate-limiting enzyme in DA production. Strikingly, this factor caused dose-dependent death of human SH-SY5Y neuroblastoma cells, a dopaminergic line. Efforts to purify the toxic activity revealed that it is a highly stable, lipophilic, and chemically unique small molecule. Evidence of a robust neurotoxic factor that selectively impacts neuronal survival in a progressive yet moderate manner is consistent with the etiology of age-associated neurodegenerative diseases. Collectively, these data suggest the potential for exposures to the metabolites of specific common soil bacteria to possibly represent a contributory environmental component to PD.

## Introduction

Neurodegenerative diseases comprise a major societal burden with increasing occurrence as our mean population age rises. The interplay between genetic predisposition and susceptibility to environmental insults lies at the core of onset and progression of several neurological diseases. For example, PD is the second-most common neurodegenerative disorder, afflicting millions of individuals worldwide. Over the course of the past decade, human genetic discoveries have driven substantial mechanistic advances in our understanding of PD [1]. Nevertheless, an inescapable fact of PD epidemiology is that over 90% of cases are of sporadic origin. Twin studies indicate that environmental influences are critical to disease onset and appear pivotal to sporadic causality [2]. Thus, while genetic analyses have substantially advanced our mechanistic understanding of PD, it is apparent that investigation into purely genetic factors will not elucidate all or even most PD incidence. Therefore, our ability to successfully reduce the frequency of PD is dependent upon knowledge about factors that render certain populations at risk.

PD affects more than 1% of the population over age 65, increasing to 4–5% in people 85-years of age. In addition to aging, one of the few established epidemiological contributors to PD appears to be a rural lifestyle. Analyses of various factors has revealed that living in rural areas, drinking well water, farming, and exposure to pesticides or herbicides may all be risk factors for developing PD [3], [4]. Toxins that cause formation of excessive reactive oxygen species, like paraquat and rotenone, as well as MPTP, all induce Parkinsonian phenotypes in animals [5]. While use of pesticides has been suggested to be partially responsible for PD in rural areas, this does not sufficiently correlate to disease prevalence, as the odds ratio for farming itself cannot be accounted for by pesticide exposure alone [6], [7].

Associated with rural living, individuals exhibit a much greater interaction with the surrounding terrestrial environment, whether by choice (e.g., occupation, avocation) or by necessity (e.g., drinking well water). A single gram of soil has been shown to contain up to 1 billion microorganisms with a predicted maximum of nearly 1 million individual microbial species. Within the order *Actinomycetales*, the ubiquitous soil bacterial genus *Streptomyces* contributes ∼6% to this total [8]. These gram positive, aerobic, organisms are responsible for producing greater than 70% of known antibiotics, in addition to a suite of other metabolites,including proteasome inhibitors. At least four characterized proteasome inhibitors are products of streptomycetes isolated from soil, including lactacystin. Impairment of the UPS as a contributory factor to sporadic PD has been suggested by a variety of studies [9]. We therefore hypothesized that enhanced exposure to these bacteria may contribute to the onset or progression of PD.

Here we show that common soil bacteria of a distinct *Streptomyces* species produce a secondary metabolite that causes neurodegeneration. Preliminary chemical characterization of the bacterial factor indicates it is a small molecular weight compound that is highly stable, hydrophobic, and chemically unique. Using a variety of mutant and transgenic lines of worms we demonstrate that the DA neurons of the nematode, *C. elegans*, exhibit enhanced vulnerability to this bacterial metabolite *in vivo*. Likewise, while other neuronal subclasses exposed to the toxin degenerated, these did not die as rapidly as DA neurons. These data were extended to human DA-producing neurons, where the bacterial metabolite also exhibited dose-dependent toxicity. Taken together, this study demonstrates that dopaminergic neurons are highly susceptible to a secondary metabolite produced by a common soil *Streptomyces* species, thereby suggesting the prospect that exposure to these strains could potentially represent a previously unreported environmental contributor to neurodegenerative disease.

## Results

### *C. elegans* DA neurodegeneration results from exposure to *S. venezuelae*

**Figure 1:**
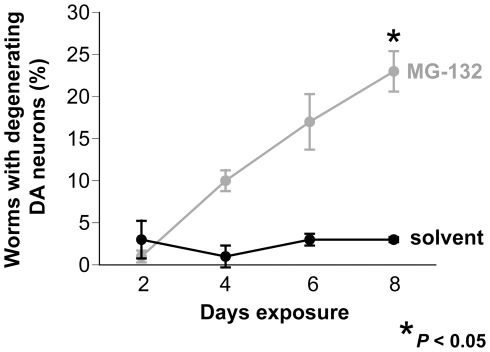
Neurodegeneration occurs in *C. elegans* following exposure to the proteasome inhibitor MG-132. An isogenic worm strain expressing GFP in DA neurons was examined for DA neurodegeneration every two days. Significant DA neurodegeneration occurred after eight days of continuous exposure to MG-132 in comparison to the solvent control (*P<0.05; ANOVA). All data in this figure are represented as mean worms with neurodegeneration +/− S.E.M.

When considering potential environmental sources of neurotoxicity associated with neurodegenerative disorders, we were intrigued by reports that described DA neurodegeneration and PD-like symptoms following direct injection of proteasome inhibitors into rat brains [10], [11]. This prompted us to initially determine if exposure to proteasome inhibitors could cause neurodegeneration in *C. elegans*. Worms expressing GFP specifically in DA neurons (P*_dat-1_*::GFP) [12] were exposed to the proteasome inhibitor MG-132 for a total of eight days and DA neurodegeneration was scored every two days, as previously described [13]. MG-132 caused a progressive loss of DA neurons (Fig. 1); after eight days of exposure, 23% of worms displayed DA degeneration compared with 3% of worms exposed to the DMSO vehicle (*P*<0.001).

Since worms displayed DA neurodegeneration in response to a purified proteasome inhibitor, we considered possible sources of naturally occurring exposures. We therefore examined the potential for secondary metabolites of common soil bacteria, *Streptomyces* species (*S. coelicolor, S. griseus*, and *S. venezuelae*), to cause DA neurodegeneration in *C. elegans*. Worms eat bacteria; *E. coli* is a standard laboratory food source used to maintain *C. elegans*. Thus, we initially attempted to directly expose these animals to *Streptomyces* spp. through feeding. However, the nematodes displayed an aversion behavior in response to *Streptomyces* spp. This is not surprising since *C. elegans* display chemosensory avoidance of unfamiliar bacteria [14]. We therefore grew each species in liquid culture and subsequently tested the conditioned growth media for neurodegenerative activity in *C. elegans*. Actinomycete metabolites are typically produced by stationary phase bacteria, therefore *Streptomyces* spp. were grown for two weeks in SYZ media (used for metabolite production) [15], [16] before cell removal and testing. *E. coli* was also grown in liquid culture as a control.

**Figure 2:**
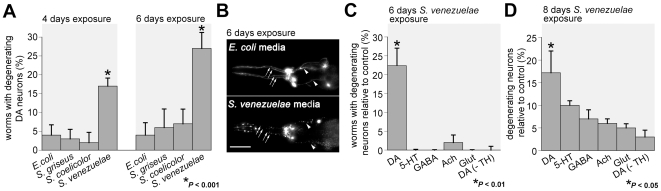
Neurodegeneration occurs in *C. elegans* following exposure to *S. venezuelae* conditioned medium. A. An isogenic worm strain expressing GFP in DA neurons was examined for DA neurodegeneration four and six days after exposure to *Streptomyces* spp. conditioned media. Significant DA neurodegeneration only occurred from exposure to *S. venezuelae* conditioned medium (*P<0.05; ANOVA). B. Photomicrographs of GFP-labeled DA neurons from *C. elegans* exposed to bacterial conditioned medium for six days. All six anterior DA neurons in *C. elegans* exhibit degenerative changes following exposure to *S. venezuelae* but not *E. coli* (control) medium (the four CEP class of DA neurons and the two ADE class of DA neurons are indicated with arrows and arrowheads, respectively). C. Populations of isogenic worm strains expressing GFP exclusively in 5-HT, GABA, ACh, Glut, and DA (+ and –TH) neuronal classes were examined for neurodegeneration. The only animals that displayed significant neurodegeneration after six days exposure to *S. venezuelae* conditioned medium were those in which the DA (+TH) neurons were analyzed (*P<0.01; ANOVA). These data were standardized against the amount of degeneration observed from exposure to *E. coli* control conditioned medium. D. At eight days of exposure to *S. venezuelae* conditioned medium, all neuronal classes examined exhibited significant degeneration (*P<0.01; ANOVA). These data were standardized against the amount of degeneration observed from exposure to *E. coli* control conditioned medium. Because each neuronal class contains varying numbers of neurons, this analysis was based on the percentage of degenerating neurons (not degenerating worms, as in Fig. 1C) to compensate for differences in total neuron numbers. Furthermore, DA neurons still exhibited significantly more degeneration than other neuronal classes (*P<0.05; ANOVA). All graphical data in this figure are represented as mean degeneration +/− S.E.M. Scale bar = 50 µM.

The conditioned bacterial media were incorporated into standard worm growth media and animals were scored for evidence of degenerative changes to DA neurons. Worms grown in the presence of *S. venezuelae* conditioned medium displayed significant and increasing degenerative changes at four and six days of exposure (Fig. 2A). For example, after six days of exposure to *S. venezuelae* conditioned medium, 27% of worms displayed DA neurodegeneration. In contrast, only 6%, 7%, and 4% of worms exposed to *S. griseus*, *S. coelicolor*, and *E. coli* media, respectively, contained degenerating DA neurons (*P*<0.001) (Fig. 2A). While the worms exposed to *S. venezuelae* medium exhibited DA neurodegenerative changes and neuronal loss (Fig. 2B), the overall lifespan and reproductive activity of these animals appeared unchanged. This was not unexpected, as DA neurons are non-essential in *C. elegans* and their loss results in only subtle behavioral changes [17].

### Enhanced vulnerability of dopamine neurons to the *S. venezuelae* factor

A distinct advantage of using *C. elegans* is the ability to discern and quantify the precise cellular complement of specific neuronal classes, permitting us to examine four other neuronal subclasses for sensitivity to the *S. venezuelae* factor. These subtypes, serotonergic (5-HT), GABAergic (GABA), cholinergic (ACh), and glutamatergic (Glut), were examined using worm strains expressing GFP specifically within these neurons using P*_tph-1_*::GFP [18], P*_unc-47_*::GFP [19], P*_unc-4_*::GFP [20] and P*_eat-4_*::GFP [21] reporter constructs, respectively. While DA neurons showed significant degeneration after four days of exposure to *S. venezuelae* conditioned medium (Fig. 2A), other neuronal classes did not exhibit significant degenerative changes at earlier ages, even at six days of continuous exposure (Fig. 2C). Specifically, ≤5% of worms displayed 5-HT, GABA, ACh, or Glut neurodegeneration, compared with 22% of worms exhibiting DA neurodegeneration at day six (*P*<0.01; data standardized with *E. coli* conditioned medium control).

When the exposure to *S. venezuelae* conditioned medium was extended to eight days, all neuronal classes exhibited some degeneration, but the DA neurons were still preferentially vulnerable compared to other neuronal classes (Fig. 2D) (*P*<0.05). Since worms possess different numbers of neurons in the various neuronal classes examined, comparative loss across different subtypes was represented in Fig. 2D by the percentage of *neurons* scored within a given subclass that were degenerating (instead of percentage of *worms* displaying at least one degenerating neuron, as reported in Fig. 2C). With this scoring method we found that, at eight days, 17% of the DA neuron population was degenerated while only 5–10% of 5-HT, GABA, ACh, and Glut neurons were degenerated (Fig. 2D). The non-dopaminergic neuronal classes all exhibited significant degeneration following eight days of exposure in comparison to the amount of degeneration exhibited after six days exposure (*P*<0.01 for these neuronal classes). These data demonstrate that continuous exposure to *S. venezuelae* metabolites causes progressive degeneration across different *C. elegans* neuronal subtypes and that DA neurons exhibit both an accelerated and enhanced vulnerability to the degenerative effect.

**Figure 3:**
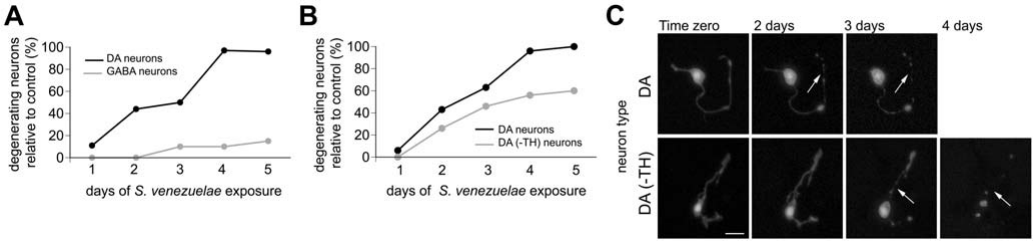
*C. elegans* neurons cultured *in vitro* display enhanced levels of degeneration in response to exposure to *S. venezuelae* conditioned medium. A. GABA neurons degenerate slowly in culture when compared to DA neurons. B. DA neurons degenerate rapidly in culture. Almost all wild-type DA neurons (+TH) are degenerated after five days of continuous exposure to the conditioned medium. A substantial proportion of DA neurons from *cat-2* mutant worms (–TH) degenerate as well. C. Photomicrographs that capture the same DA neurons (+ and –TH) over the course of several days exposure to the *S. venezuelae* factor. There is one less image for DA+ neurons because this neuron had degenerated by day four and was not visible. Arrows depict degeneration in cell processes. All graphical data in this figure are represented as mean degeneration relative to *E. coli* conditioned medium controls (control degeneration was always <2%). Scale bar = 5 µM.

The anatomical placement of the different neuronal subtypes within the intact nematode body plan may partially account for differential access of toxin to select neurons. Moreover, it is possible that the *C. elegans* cuticle impedes entry of the *S. venezuelae* metabolite *in vivo*. Therefore, we generated primary cultures of distinct *C. elegans* neuron subtypes to determine if significant enhancement in degeneration of various neuronal subtypes occurs *in vitro*. A modest increase in the effect of the *S. venezuelae* metabolite was observed, with the degeneration of 10% of GABA neurons (vs. 6% *in vivo*) and 15% ACh neurons scored (vs. 7% *in vivo*) (Fig. 3A). However, as described below, DA neurons exhibited a much higher enhancement in degeneration when cultured. Thus, it appears that the cuticle might provide a partial barrier from the degenerative factor, but that DA neurons still retain their selective vulnerability when cultured, suggesting that an intrinsic factor within DA neurons is associated with enhanced degeneration (Fig. 2C, 2D vs. 3A ).

### The presence of dopamine exacerbates neurodegeneration

Some DA toxins, such as 6-hydroxydopamine (6-OHDA), are selective because they are substrates for the DA transporter (DAT). To explore the possibility that the *C. elegans* DA transporter, DAT-1 might facilitate entry of the *S. venezuelae* factor, *dat-1* mutant worms expressing GFP in DA neurons (*dat-1*; P*_dat-1_*::GFP), which are deficient for DAT-1 [13], [22], were examined for neurodegeneration in response to the factor. After six days of exposure to *S. venezuelae* conditioned medium there was no significant difference in DA neurodegeneration between WT and *dat-1* worms (24±1.3% vs. 29±4.4% worms with DA neurodegeneration, respectively). We therefore conclude that the neurotoxic *S. venezuelae* metabolite is not specifically entering DA neurons through the DAT-1 transporter.

Since DA neurons degenerate more readily than other neuronal classes, we hypothesized that the presence of DA itself might enhance the neurodegeneration associated with exposures. To examine this, we exposed *cat-2* (*e1112*) mutant worms [23] to *S. venezuelae* conditioned medium. *cat-2* worms express reduced levels of TH, the rate-limiting enzyme in the production of DA, and as a result, contain only 40% of wild-type DA levels [24]. After six days of exposure, populations of *cat-2* worms (-TH) displayed substantially less degeneration (1%) than wild-type worms with normal levels of DA synthesis (22%) (*P*<0.001; Fig. 2C). Notably, when examining the comparative loss of individual neurons after eight days of exposure to *S. venezuelae* medium (Fig. 2D), only 3% of DA (-TH) neurons within *cat-2* worms exhibited DA neurodegeneration compared with 17% of DA neurons in wild-type worms (*P*<0.01). Therefore, the presence of DA appears to provide a sensitized cellular milieu for the *S. venezuelae* factor that exacerbates neurodegeneration.

We also examined the effect of the *cat-2* mutant *in vitro*. Wild-type and *cat-2* mutant neurons were cultured in the presence of the *S. venezuelae* factor and scored for rates of degeneration. The percentage of degenerated neurons was standardized against *E. coli* conditioned medium (where 0–1.5% of neurons degenerated). Notably, in culture, DA neurons degenerate faster when exposed to the *S. venezuelae* factor; strikingly, 100% of the wild-type DA neuron population (+TH) displayed degenerative changes at day five of continuous exposure while only 60% of the *cat-2* DA neurons (-TH) also displayed degenerative changes (Fig. 3B, 3C). To determine if conditioned media from all three *Streptomyces* spp. examined in our study could cause significant degeneration in culture, wild-type DA neurons were exposed to *S. griseus* and *S. coelicolor* conditioned media at the same concentration as *S. venezuelae* (0.5%). No degeneration (0%) was observed in the cultured DA neurons, even after five days of exposure. Thus, the enhanced degenerative effect observed in DA neurons is highly specific to the *S. venezuelae* factor.

### The *S. venezuelae* neurodegenerative factor inhibits the UPS

**Figure 4:**
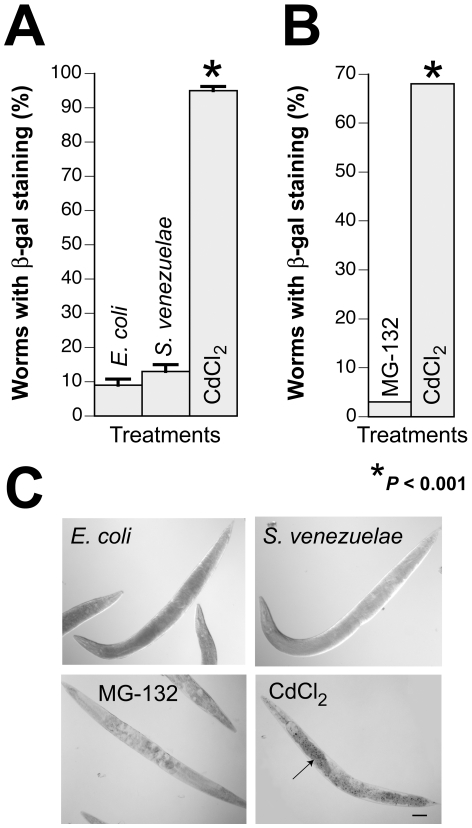
The *S. venezuelae* factor does not enhance expression of *hsp-16*, a small heat shock protein. LacZ expression is driven from the *hsp-16* promoter in an isogenic worm strain. X-gal staining was used to examine expression of β-galactosidase (β-gal) in these animals. A. *hsp-16* expression occurs in response to specific stressors, such as CdCl_2_. In contrast, *hsp-16* expression is minimal within populations of *C. elegans* exposed to *S. venezuelae* or *E. coli* conditioned media for six days (*P<0.001; ANOVA). These data are represented as mean worms positively stained with X-gal +/− S.E.M. B. Worms exposed to the proteasome inhibitor MG-132 for 6 days display minimal expression of *hsp-16*, in contrast to the CdCl_2_ control (*P<0.001; Fisher Exact Test). C. Photomicrographs depicting representative worms exposed to *E. coli* or *S. venezuelae* conditioned medium, MG-132, or CdCl_2._ *C. elegans* exposed to CdCl_2_ exhibited prominent hypodermal induction of β-gal (arrow). Scale bar = 100 µM.

Several mechanisms associated with intracellular stress and neurodegeneration could be influenced by the *S. venezuelae* factor. For example, this toxin might trigger a generalized chaperone-mediated stress response within *C. elegans* cells. HSP-16 is a *C. elegans* homolog of the hsp16/hsp20/alphaB-crystallin family of heat shock proteins. In P*_hsp-16_*::lacZ worms, expression of β-galactosidase is driven by the *hsp-16* promoter following exposure to specific chemical or physical stressors [25], [26]. To examine whether the *S. venezuelae* metabolite activates small heat shock proteins, P*_hsp-16_*::lacZ worms were exposed to *E. coli* and *S. venezuelae* conditioned media (Fig. 4). Neither conditioned medium induced significant β-galactosidase expression in P*_hsp-16_*::GFP worms (Fig. 4A, C). In comparison, most worms exposed to an established general stressor, CdCl_2_, expressed high levels of β-galactosidase (*P*<0.001; Fig. 4A), which was evident within the hypodermis (Fig. 4C). Worms were also treated with the proteasome inhibitor MG-132, which did not induce significant β-galactosidase expression (Fig. 4B, C).

**Figure 5:**
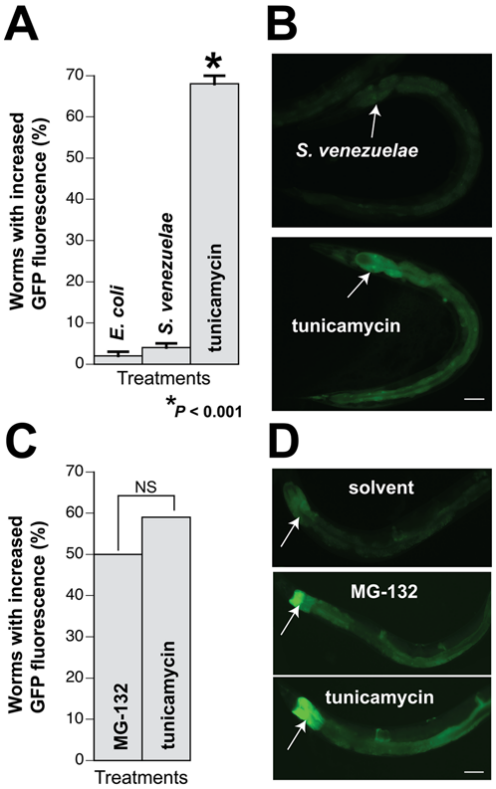
The UPR is not activated from exposure to the *S. venezuelae* factor. Isogenic worms expressing GFP under control of *hsp-4* (homolog of mammalian BIP) were exposed to conditioned media or MG-132 for six days and then examined for GFP expression. A. Significantly higher levels of GFP fluorescence were observed within populations of *C. elegans* exposed to the positive control, tunicamycin, but not within populations animals of exposed to *S. venezuelae* or *E. coli* conditioned media for six days (*P<0.001; ANOVA). These data are represented as mean worms with increased GFP fluorescence +/− S.E.M. B. Representative images of whole worms exposed to *S. venezuelae* conditioned medium or tunicamycin. The worm exposed to tunicamycin exhibited stronger fluorescence, particularly in the region of the intestine proximal to the pharynx (arrow). Scale bar = 100 µM. C. MG-132, a proteasome inhibitor, activates the UPR at a level that is non-significantly different from tunicamycin (*P* = 0.653; Fisher Exact Test). D. Representative images of magnified portions of *C. elegans* exposed to the DMSO solvent (required for both MG-132 and tunicamycin), MG-132, and tunicamycin. Arrows indicate region of intestine proximal to the pharynx in all three animals. Scale bar = 50 µM.

The unfolded protein response (UPR) is another common mechanism associated with cellular stress and PD [27]. We examined the possibility that the *S. venezuelae* factor up-regulated the UPR by exposing worms expressing an established UPR reporter, P*_hsp-4_*::GFP, to conditioned medium [28]. *C. elegans* HSP-4 is homologous to the mammalian ER chaperone, BiP, and its transcription is prominently induced in the worm intestine in response to ER stress. We determined that the *S. venezuelae* neurodegenerative factor did not induce *hsp-4*, as higher levels of *hsp-4*::GFP were observed in only 2% and 5% of worms exposed to *E. coli* and *S. venezuelae* conditioned media, respectively (Fig. 5A, B). In contrast, 68% of control animals exposed to an established UPR-inducer, tunicamycin, exhibited robust *hsp-4*::GFP expression (*P*<0.001; Fig. 5A, B). Worms exposed to MG-132 displayed an increased level of GFP fluorescence that was comparable to tunicamycin (*P* = 0.653; Fig. 5C, D). These data are consistent with a previous study wherein canine kidney cells exposed to MG-132 displayed increased BiP expression [29].

**Figure 6:**
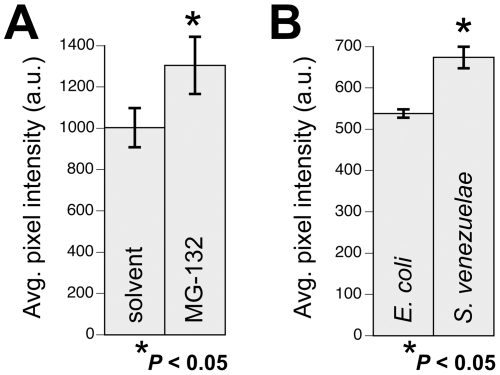
Ubiquitin-related degradation is impaired in *C. elegans* DA neurons following exposure to the *S. venezuelae* factor. A. Isogenic worms expressing a CFP::CL-1 degron fusion protein within DA neurons were exposed to the proteasome inhibitor MG-132. Mean pixel intensity of CFP fluorescence is significantly higher when the proteasome in inactived (*P<0.05; Fisher Exact Test). B. CFP fluorescence is also significantly higher following exposure to *S. venezuelae* conditioned medium when compared to *E. coli* medium (*P<0.05; Fisher Exact Test). All data in this figure are represented as mean pixel intensities [in arbitrary units (a.u.)] +/− S.E.M.

Another candidate cellular mechanism potentially modulated by the *S. venezuelae* neurodegenerative factor is the UPS. We adapted an *in vivo* fluorescence-based assay for proteasome inhibition [30] by expressing a ubiquitination signal (CL-1, also referred to a “degron”) fused to CFP within the DA neurons of *C. elegans* (P*_dat-1_*:: CFP::CL-1). This CFP::CL-1 reporter functions as a biomonitor for ubiquitin-related degradation whereby CFP fluorescence levels are low when the protein is degraded (i.e., UPS is active) and the fluorescence much higher when the protein is not degraded (UPS activity is functionally impaired). When CL-1::CFP worms are exposed to MG-132, we observed a 25% increase in DA neuron fluorescence in comparison to worms exposed to the DMSO solvent (Fig. 6A). Notably, exposure to *S. venezuelae* conditioned medium also resulted in a significant, 18%, increase of CFP fluorescence when compared with worms exposed to *E. coli* conditioned medium (*P*<0.05) (Fig. 6B). Based on our initial mechanistic studies, the *S. venezuelae* toxin does not elicit a generalized chaperone-mediated stress response or upregulate the UPR, but it does block degron degradation. Therefore, we conclude that the *S. venezuelae* conditioned medium contains a neurotoxic activity that blocks the UPS.

### Human DA cells are sensitive to the *S. venezuelae* factor

**Figure 7:**
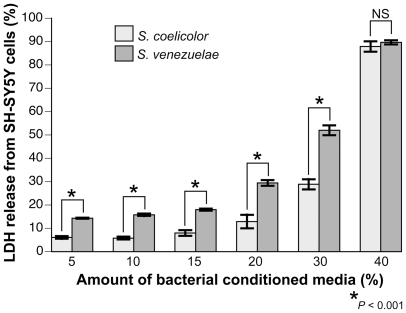
Human SH-SY5Y neuroblastoma cells exhibit cell death following exposure to *S. venezuelae* conditioned medium. Cell death was measured using LDH release; it was significantly enhanced when cells were exposed to *S. venezuelae* in comparison to *S. coelicolor*. This experiment was performed three times, in duplicate (n = 6) (*P<0.001; ANOVA). These data depict one independent experiment that is representative of the others, whereby mean LDH release is displayed as +/− S. D.

While the nematode model is an excellent system that can provide preliminary toxicological and mechanistic insights, ultimately it is important that these findings translate to human biology. Human SH-SY5Y neuroblastoma cells can synthesize DA, and are commonly used as a cellular model of PD. These cells were exposed to conditioned media from *S. venezuelae* or *S. coelicolor*, the latter of which did not cause significant neurodegeneration in *C. elegans* neurons. Following 48 hours of exposure, cell viability was measured by release of the intracellular enzyme, lactate dehydrogenase (LDH). The extent of cell death was significantly higher when SH-SY5Y cells were exposed to conditioned media from *S. venezuelae* compared to *S. coelicolor* at most concentrations tested (*P*<0.001; Fig. 7), until the amount of bacterial conditioned medium reached 40% within the cell culture media. These data confirm that the *S. venezuelae* neurotoxic activity characterized in the *C. elegans* model also has the capacity to cause degeneration of human DA-producing neurons.

### Characterization of the neurodegenerative factor

**Figure 8:**
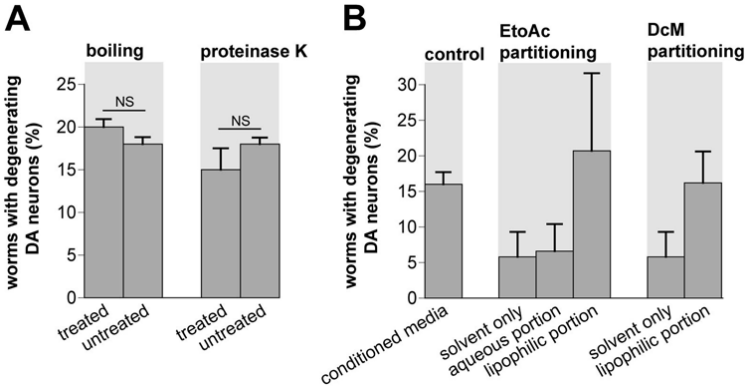
The *S. venezuelae* neurodegenerative factor is lipophilic and stable. A. Boiling conditioned media for 30 minutes or digestion with proteinase K had no discernable effect on the neurodegenerative effect of the *S. venezuelae* factor. All data in this figure are represented as percentage of mean worms exhibiting DA neurodegeneration. B. Partitioning with ethyl acetate (EtoAc) resulted in activity separation within the organic but not the aqueous phase (compare ethyl acetate solvent only with aqueous portion). Further partitioning with dichloromethane (DcM) also resulted in the activity separating in the lipophilic portion. In all cases, extracts were dried and resolubilized with ethyl acetate (solvent only control), as it is non-lethal to *C. elegans*. The amount of DA neurodegeneration observed in the conditioned media control was lower than previously reported because 34% less factor was used in each experiment (to ensure final ethyl acetate concentrations were below 1.5%).

We have initiated the molecular characterization and isolation of the *S. venezuelae* metabolite and it possesses a unique suite of chemical properties. We heated the *S. venezuelae* conditioned media at 100°C for 30 minutes before treatment of *C. elegans* DA neurons. Boiling had no effect on the neurodegenerative activity of the factor. Specifically, 20% vs. 18% of worms displayed DA neurodegeneration after six days exposure to heat-treated and untreated factor, respectively (Fig. 8A). Likewise, proteinase K digestion did not alter the activity; 15% worms exhibited DA neurodegeneration after exposure to the treated *S. venezuelae* factor (vs. 18% of worms exposed to untreated factor; Fig. 8A). Thus, the *S. venezuelae* factor appears to be a highly stable and robust bacterial metabolite, characteristically distinct from known proteasome inhibitors.

We have begun chemical characterization of the neurotoxic metabolite and have tested the product from each stage of the purification process for DA neurodegeneration using *C. elegans*. The *S. venezuelae* metabolite is soluble in water as demonstrated by the presence/activity in conditioned medium and can be partitioned from water into either ethyl acetate or dichloromethane (Fig. 8B), suggesting that the molecule has both lipophilic and hydrophilic portions. When passed through a silica column to further purify the factor, it partitions into the glyco- and polar lipid fractions. That is, very little neurodegenerative activity was recovered in the chloroform or first two acetone fractions; however, the neurodegenerative factor was recovered in the last two acetone and methanol fractions. The methanol fraction showed a 6.5-fold increase in activity compared to *E. coli* conditioned medium, again, suggesting a molecule with both lipophilic and hydrophilic portions [31].

Lipopolysaccharide (LPS) is an endotoxin produced by gram-negative bacteria that is associated with a reduced number of DA neurons in rats when exposure occurs during the prenatal period [32], [33]. *S. venezuelae*, however, is a gram-positive bacterium and these microbes are classified, in part, by an absence of LPS. Therefore, taken together with the fact that LPS is insoluble in dichloromethane, it is very unlikely that the *S. venezuelae* neurodegenerative factor contains LPS.

To further purify this toxin, we used thin-layer chromatography (TLC) of the methanol fraction and observed four distinct bands. The bands were excised, eluted, and analyzed by gas chromatography/mass spectrometry, which produced a chromatogram that contained two related compounds. The peak shape of these compounds is consistent with the polar nature of the molecule and suggests an ionic functional group (possibly an underivatized hydroxyl or amino functional group). Mass spectral analysis of these compounds indicates a putative molecular weight of 180; each spectrum contains a base peak of m/z 180, a second predominate ion of m/z 70 and a fragmentation pattern indicative of a hydrocarbon chain as a component of the parent molecule. Of the five strong candidates for the molecular formula of the molecule (*i.e.*, molecular weight 180; C_13_H_24_, C_12_H_20_O, C_11_H_20_N_2_, C_11_H_16_O_2_, C_10_H_16_N_2_O), the isotope pattern fits best with C_10_H_16_N_2_O. Based on standard mass spectra analysis algorithms, the factor likely contains an aromatic or heterocyclic ring and a hydrocarbon chain (C_5_H_11_ or C_6_H_13_). While outside the scope of this work, our continuing studies involve the isolation and structural elucidation of the *S. venezuelae* neurodegenerative factor. Once we have identified the chemical structure of the factor, this will assist with a further understanding of the mechanism by which it is causing neurodegeneration in *C. elegans* or SH-SY5Y cells, and will facilitate development of *in vivo* mammalian models.

## Discussion

Onset and progression of PD appears to converge at the intersection between environmental influences, intrinsic DA metabolism, and accumulation of misfolded proteins to yield enhanced susceptibility to neurodegeneration over the course of time. Increasing evidence supports deficits in, or inhibition of protein degradation and clearance pathways by either genetic or environmental factors as impacting neurodegeneration [9]. Indeed, a balance exists between proteasome function and autophagy or lysosomal-based degradation as complementary and compensatory mechanisms to mediate clearance of misfolded proteins [34], [35]. It is intriguing to consider that even mild toxin-mediated UPS inhibition may combine with genetic deficit in select autophagy genes to yield enhanced susceptibility to neurodegeneration. Support for a “multiple hit” hypothesis for induction of neurodegeneration in PD has recently come from an elegant study indicating that increased cytosolic DA levels influence neurotoxicity through generation of oxidized metabolites that interact with α-synuclein, an effect that is blocked by calcium channel blockers [36]. We previously showed that overexpression of the *C. elegans* TH homolog, CAT-2, in nematode DA neurons also led to increased neurodegeneration, an effect that was blocked by 3-iodotyrosine treatment (13). Given the substantial neuroprotective impact of TH mutants on the toxicity caused by the *Streptomyces* metabolite in *C. elegans*, it is interesting to speculate this environmental factor may represent an added multifactoral component in DA neurodegeneration.

Several prior studies have reported selective loss of DA neurons after systemic administration of lactacystin or PSI to rodents [10], [37], [38], but other investigators have had difficulty reproducing these results [39], [40]. Direct administration of either of these agents into the brain, however, leads to a reliable and consistent progressive degeneration of dopaminergic neurons [11], [41]. It has also recently been demonstrated that genetic depletion of the 26S proteasome subunit results in protein inclusions and neurodegeneration in mice [42]. In this latter report, conditional knockout in targeted brain neurons of mice clearly demonstrates a role for proteasome function in neuronal homeostasis and survival. This is significant to consider in the context of our findings showing that our neurotoxic factor inhibits proteasome activity of *C. elegans* DA neurons. Although a putative route of environmental exposures to humans remains undefined, the small size and stability of the bacterial metabolite we have identified suggests a robust neurotoxic capacity. Additional work to define the intracellular target(s) of this toxin will likely reveal more about the mechanism underlying neurodegeneration.

While non-pathogenic bacteria have not been previously implicated in PD-associated degeneration, an earlier report [43] demonstrated that mice injected with *Nocardia asteroides*, a human pathogen also of the order *Actinomycetales*, developed PD-like symptoms and responded to levodopa treatment. Follow-up studies showed that *N. asteroides* strain GUH-2 was neuroinvasive in the brains of mice [44], [45] and monkeys [46] and that infection resulted in apoptotic death of DA neurons associated with proteasome inhibition [47], [48]. Another nocardial strain (GAM-5) has also been shown to induce impaired movements and similar pathological features in mice following sublethal infection [49]. Characterization of culture filtrates from strain GUH-2 indicated that a non-lipid, small molecular weight, secreted substance may be responsible for DA-depleting effects [50]. While the chemical profile of the *S. venezualae* neurotoxic factor is distinct from the nocardial metabolite, taken together, these reports emphasize the potential impact of bacterial exposures on neurodegeneration. The prevalence of PD argues against pathogenic bacterial infection as a major contributory element in disease etiology. Nevertheless, in the context of our findings with more common non-pathogenic bacterial species, a prospective role for bacterial metabolites as environmental effectors of neurotoxicity is understudied and should perhaps be revisited. Moreover, the progressive nature of PD is reflected in the moderate and age-associated gradated degeneration we observed in response to the *Streptomyces* neurotoxic metabolite in *C. elegans* and warrants further investigation in mammalian *in vivo* models. Importantly, without knowing the precise identity of the factor(s) produced by these bacteria, or the concentrations at which they are toxic to humans compared to those applied in this report, any correlations to PD pathogenesis are indirect and should be taken with caution, as this remains to be resolved through future studies.

From an epidemiological perspective, such studies incite obvious questions regarding the source or extent of exposures. A rural lifestyle, drinking well water, farming, and exposure to pesticides or herbicides may all be risk factors for developing PD [3], [4]. Systematic evaluation of occupational, domestic, and recreational exposures to actinomycete-rich soil or adjacent well water sources may reveal unforeseen correlates to PD. In this regard, surveys of strain-specific effects, chronic vs. acute exposure paradigms, and community-based epidemiological analyses are all required to better define the impact of bacterial sources of neurotoxicity. Further understanding the combined impact of such exposures with genetic susceptibility will serve to better define therapeutic interventions. Moreover, the prospect that bacterial metabolites could represent putative biomarkers for disease susceptibility could serve to greatly accelerate future diagnosis and refine risk assessment.

## Materials and Methods

### Nematode strains

Nematodes were maintained using standard procedures [51]. Strain UA30 (*baEx30*) consists of P*_dat-1_*::GFP in *dat-1* knockout worms [RM2702 (*ok157*), a functional null] [13]. Additional strains, as described below, were obtained from the *Caenorhabditis* Genetics Center. These strains include N2 Bristol, SJ4005 [*zcIs4*(*hsp-4*::GFP)] (integrated P*_hsp-4_*::GFP strain) [28], CB1112 [*cat-2*(*e1112*)II] [23], BY200 *vtIs1*[P*_dat-1_*::GFP + pRF4(*rol-6*(*su1006*))] (an integrated P*_dat-1_*::GFP strain) [13], EG1285 [*lin-15(n765ts);oxIn12*] (an integrated *unc-47*::GFP strain) [19], GR1366 *mgIs42*[*tph-1*::GFP + pRF4(*rol-6*(*su1006*))] (an integrated P*_tph-1_*::GFP strain) [18], DA1240 *adIs1240*[*lin-15*((+) *eat-4*::GFP) *lin-15B*(*n765*) X] (an integrated P*_eat-4_*::GFP strain) [21], and NC300 *wdIs5*[(*dpy-20*(*e1282*) IV] (an integrated P*_unc-4_*::GFP strain) [20], PC72 [48.1C and pRF4[rol-6(*su1006*))] (an integrated P*_hsp-4_*::lacZ strain) [25].

For the generation of *cat-2*; P*_dat-1_*::GFP animals, P*_dat-1_*::GFP males were crossed to *cat-2* hermaphrodites [23]. F_1_ hermaphrodites expressing GFP were allowed to self and F_2_ worms were singled onto plates. The genomic region encompassing the *cat-2* genetic lesion (stop codon) was sequenced from worm lines where 100% of the F_3_ generation expressed GFP (sequencing repeated three additional times).

To construct P*_dat-1_*::CFP::CL-1 transgenic worms, pPD133.48, a CFP containing plasmid (gift from Andy Fire) was used as a template to amplify CFP::CL-1 using a CFP specific forward primer and a reverse primer that incorporated the last few nucleotides of CFP and the entire CL-1 sequence [30] (sequences available upon request). The CFP::CL-1 fusion was cloned into a Gateway pDONR221 vector (Invitrogen) and then recombined into pDEST-DAT1 [13]. 10 µg each of P*_dat-1_*::CFP::CL-1 plasmid DNA and an *unc-119* rescuing vector (pDP#MM016B) [52] were co-introduced into *unc-119* worms by biolistic bombardment using a Bio-Rad Biolistic PDS-1000/He particle delivery system [53]. A rescued, stable (*unc-119* ^+^) line expressing CFP in DA neurons was integrated using UV-irradiation in a Spectroline UV crosslinker at 254 nm at 200 mJ/cm^3^. Multiple integrated lines were compared and a representative line was examined for sensitivity to MG-132, a proteasome inhibitor. This line [UA96 [*baIn16*; *unc-119* (+) P*_dat-1_*::CFP-CL-1) *unc-119* (ed3)] was out-crossed three times and then used for all subsequent experiments.

### *C. elegans* neurodegeneration assays

Three replicates of 30–40 worms were analyzed using a Nikon E800 compound microscope with epi-fluorescence using an Endow GFP filter cube (Chroma). Neurons were analyzed for missing, shortened or blebbing processes, as well as rounding or loss of cell bodies, as previously described [13]. Worms were scored as having normal or wild-type neurons when none of these abnormalities were present in any neurons. When neuron loss was compared across treatments, degeneration of neurons in whole worm populations was reported (e.g., DA neuron analysis). However, when degeneration levels across different types of neurons were compared to each other (for example, comparing DA neurons to GABA neurons), percentages of degenerating neurons were reported because varying numbers of neurons were analyzed in the subclasses (21 ACh, 6 DA, 26 GABA, 23 Glut, and 6 5-HT neurons). Statistics were performed using ANOVA.

### *C. elegans* exposure to proteasome inhibitors

L4 stage larvae of P*_dat-1_*::GFP worms were grown on *E. coli* (strain OP50) seeded NGM plates. MG-132 (EMD Biosciences) was incorporated into the media at a concentration of 50 µM in DMSO. Worms were transferred to new plates containing fresh MG-132 every two days. DA neurons were assayed for either degeneration or pixel intensity changes (CFP::CL-1) at time points described in the results section and compared to worms exposed to DMSO only.

### *C. elegans* assay for generalized stress response

L4 stage P*_hsp-16_*::lacZ worms [25] were exposed to 25 µl/ml conditioned media from either *S. venezuelae* or *E. coli*, 50 µM MG-132, 0.2% DMSO as a solvent control, or 0.03 mg/ml CdCl_2_ [26] for six days; animals were transferred to fresh plates (with treatment) every two days. Approximately 300 P*_hsp-16_*::lacZ worms/condition were fixed and stained using standard techniques [54]. Fixed worms were scored for lacZ staining under a Nikon compound microscope. Worms exhibiting X-gal stain were scored as “stressed” while worms without staining were scored as “unstressed”. Statistics were performed using ANOVA or Fisher Exact Test, as described in the figure legend.

### *C. elegans* ER stress assay

Approximately 300 worms per treatment (25 µl/ml conditioned media from either *S. venezuelae* or *E. coli*, 50 µM MG-132, 0.2% DMSO as a solvent control, or 0.03 µg/ml tunicamycin) were assessed for upregulation of *hsp-4* via GFP intensity analysis [28]. L4 animals were transferred to fresh plates (with treatment) every two days. Worms were exposed to the treatments for 6 days before analysis. Fluorescent levels were visually assessed using a Nikon fluorescent dissecting scope. If worms expressed GFP throughout the length of the intestine (where *hsp-4* is expressed), and displayed increased expression in the region of intestine proximal to the pharynx, they were scored as having increased GFP fluorescence; otherwise, when GFP levels were low (very little expression in the intestine), they were scored as having basal GFP fluorescence. Statistics were performed using ANOVA or Fisher Exact Test, as described in the figure legend.

### *C. elegans in vivo* degron analysis

L4 stage worms were exposed to 50 µM MG-132 in DMSO for 2 days before fluorescent analysis. We empirically determined that 2 days of exposure resulted in proteasome inhibition without corresponding to DA neurodegeneration (see Fig. 1). Three replicates of 30 P*_dat-1_*::CFP::CL-1 worms were analyzed using a Nikon E800 epi-fluorescent microscope equipped with a CFP filter cube (Chroma). The average pixel intensity was measured for every CEP neuron/worm using Metamorph software and these values were compared between the solvent control and the proteasome inhibitor treatment using the Fisher Exact Test. Similarly, 30 P*_dat-1_*::CFP::CL-1 worms were exposed to *S. venezeulae* excretions or *E.coli* (control) conditioned media for 3 days before analysis, as described above. Statistics to compare across all treatments was performed using ANOVA.

### Growth of *Streptomyces* spp

We examined three common soil bacteria from the genus *Streptomyces* [*S. venezuelae* USDA-ARS ISP-5230, *S. griseus* USDA-ARS B-2165, and *S. coelicolor A3* (2)]. Spores were rehydrated at a density of 1 ×10^6^ in 1.2 L SYZ media. Because secondary metabolites are excreted in response to different factors in the environment [15], we tested different growth media to optimize production of the neurodegenerative factor. *Streptomyces* species were grown in nematode growth media (NGM), NGM with 0.15 M sorbitol, nutrient broth (NB), SYZ broth (soluble starch, yeast extract, NZ amine) without XAD resin [16], or brain heart infusion (BHI) medium for 2 weeks at 30°C. Cells were removed by centrifugation at 10,000 x *g* in a Sorvall for 10 minutes and supernatants were filtered through two filters, a 0.45 µm PES vacuum filtration unit, followed by a 0.22 µm PES unit. After filtration, conditioned media was dispensed into single use aliquots and frozen for subsequent use. Exposures consisted of 25 µl/ml conditioned media in each plate; L4 worms were transferred to fresh plates (with treatment) every two days. After six days of continuous exposure to the conditioned medium SYZ medium, 27% of worms displayed DA neurodegeneration, while only 6%, 4%, 7%, and 6% of worms exhibited DA neurodegeneration following exposure to NGM, NGM with sorbitol, NB and BHI media, respectively. Therefore, SYZ medium was used for all assays.

### *C. elegans* primary neuron cell culturing

Embryonic cell isolation and cell culture conditions were performed as previously described [55]. After plating cells on culture plates, cells were allowed to differentiate at 23°C for 48 hours before bacterial conditioned media were administered (*E. coli*, *S. venezuelae*, *S. coelicolor*, or *S. griseus*) at a final concentration of 0.5% within the cell culture media. The L-15 culture media with bacterial media was replaced every two days. Cells were analyzed on a Leica Confocal microscope. 50–90 cells per treatment condition were monitored and scored for degeneration daily, for 7–9 days.

### Mammalian cell culture methods

SH-SY5Y cells were maintained on Corning dishes in Dulbecco's modified Eagle's medium (DMEM) supplemented with 10% fetal bovine serum, 10 U/ml penicillin, 100 µg/ml streptomycin. Cells were grown in a humidified atmosphere containing 5% CO_2_. For treatment, approximately 150,000 cells were plated in each well of 24-well plates. Twenty-four hours after plating, SH-SY5Y cells were treated with various concentrations (5–40%) of *Streptomyce*s spp. conditioned media in serum-free culture medium for 48 h. The release of LDH into the medium was used as a quantitative measurement of cell viability and was carried out as previously described [56]. The level of LDH release following exposure to *S. coelicolor* conditioned media was used to normalize the levels of LDH release observed following *S. venezuelae* treatment to standardize the values. Experiments were performed in triplicate and repeated three times. Statistics were performed using ANOVA.

### Solvent extraction of the conditioned media

Filtered culture broths were extracted with an equal volume of ethyl acetate or dichloromethane using a separatory funnel. The mixture was gently shaken and the phases allowed to separate. The ethyl acetate or dichloromethane layer was collected and the process was repeated two more times. The organic phases were pooled and dried [31]. The dried extract were weighed and resuspended (3 mg/ml) in ethyl acetate for worm DA neurodegeneration assays, which were performed blinded. In these assays, 15 µl/ml of partially purified *S. venezuelae* medium or 15 µl/ml ethyl acetate (solvent control) was added to Petri dishes for a final concentration of 45 µg/ml in the agar nematode media. L4 worms were exposed to fresh bacterial extract or solvent every two days and DA neuron analysis was performed after six days of exposure.

### Chemical characterization of *S. venezuelae* factor

Two different treatments were used to examine the stability of the *S. venezuelae* factor: 1) conditioned medium was boiled for 30 minutes, and 2) conditioned medium was exposed to 0.5 mg/mL proteinase K for 1 hr at 65°C prior to heat inactivation of the enzyme at 95°C for 10 minutes. Both forms of treated *S. venezuelae* conditioned medium were added to NGM plates and L4 stage worms were grown in the presence of the factor for six days. DA neurodegeneration in *C. elegans* was assayed as described previously. Untreated *S. venezuelae* conditioned medium exposure was used as a positive control.

Silicic acid column chromatography was used to further partition the dichloromethane soluble fraction. A 2.5 cm diameter column packed with 16 g of silica gel (Fisher Scientific, 70–230 µm and 60 A, total volume 18 cm^3^) was used for separation. A flow rate of ∼5 ml/min was used for elution. Dried extracts of filtered culture broths were dissolved in a minimum volume of dichloromethane and added to the column. Fractions were eluted from the column in 10, 4×10, and 10 column volumes of chloroform, acetone, and methanol, respectively, yielding a total of six fractions. Fractions were dissolved in ethyl acetate at 1 mg/ml and tested for neurodegenerative activity in *C. elegans* as described above, with the highest levels being detected in the methanol fraction (which was only partially soluble in ethyl acetate). This fraction was loaded onto a silica TLC plate and separated using a chloroform∶acetone∶methanol∶acetic acid∶water (10∶4∶2∶2∶1) solvent mixture. After the solvent had traversed 95% of the plate height, the plate was removed, dried, and the position of the compound bands determined. Four discrete bands were observed; each was removed from the plate and the compounds present eluted from the silica with methanol. The solvent was dried and the sample resuspended in chloroform and injected into an Agilent 6890 gas chromatograph fitted with a DB-5 fused-silica column and an Agilent 5893 Mass Selective Detector operated in scan mode. Ion impact spectra of individual compounds were generated and interpreted using standard organic chemical concepts.

## References

[1] Dawson TM, Dawson VL (2003). Molecular pathways of neurodegeneration in Parkinson's disease.. Science.

[2] Tanner CM (2003). Is the cause of Parkinson's disease environmental or hereditary? Evidence from twin studies.. Adv Neurol.

[3] Priyadarshi A, Khuder SA, Schaub EA, Priyadarshi SS (2001). Environmental risk factors and Parkinson's disease: a metaanalysis.. Environ Res.

[4] Costello S, Cockburn M, Bronstein J, Zhang X, Ritz B (2009). Parkinson's disease and residential exposure to maneb and paraquat from applications in the central valley of California.. Am J Epidemiol.

[5] Tanner CM (1992). Occupational and environmental causes of parkinsonism.. Occup Med.

[6] Gorell JM, Johnson CC, Rybicki BA, Peterson EL, Richardson RJ (1998). The risk of Parkinson's disease with exposure to pesticides, farming, well water, and rural living.. Neurol.

[7] Firestone JA, Smith-Weller T, Franklin G, Swanson P, Longstreth WT (2005). Pesticides and risk of Parkinson disease: a population-based case-control study.. Arch Neurol.

[8] Janssen PH (2006). Identifying the dominant soil bacterial taxa in libraries of 16S rRNA and 16S rRNA genes.. Appl Environ Microbiol.

[9] Cook C, Petrucelli L (2009). A critical evaluation of the ubiquitin–proteasome system in Parkinson's disease.. Biochim Biophys Acta.

[10] McNaught KS, Perl DP, Brownell AL, Olanow CW (2004). Systemic exposure to proteasome inhibitors causes a progressive model of Parkinson's disease.. Ann Neurol.

[11] Sun F, Anantharam V, Zhang D, Latchoumycandane C, Kanthasamy A (2006). Proteasome inhibitor MG-132 induces dopaminergic degeneration in cell culture and animal models.. NeuroTox.

[12] Nass R, Hall DH, Miller DM, Blakely RD (2002). Neurotoxin-induced degeneration of dopamine neurons in *Caenorhabditis elegans*.. Proc Natl Acad Sci U S A.

[13] Cao S, Gelwix CC, Caldwell KA, Caldwell GA (2005). Torsin-mediated protection from cellular stress in the dopaminergic neurons of *Caenorhabditis elegans*.. J Neurosci.

[14] Zhang Y, Lu H, Bargmann CI (2005). Pathogenic bacteria induce aversive olfactory learning in *Caenorhabditis elegans*.. Nature.

[15] Deimain AL, Fang A, Scheper T (2000). The Natural Functions of Secondary metabolites.. Advances in Biochemical Engineering/Biotechnology.

[16] Park YC, Gunasekera SP, Lopez JV, McCarthy PJ, Wright AE (2006). Metabolites from the marine-derived fungus *Chromocleista* sp. isolated from a deep-water sediment sample collected in the Gulf of Mexico.. J Natural Products.

[17] Sawin ER, Ranganathan R, Horvitz HR (2000). *C. elegans* locomotory rate is modulated by the environment through a dopaminergic pathway and by experience through a serotonergic pathway.. Neuron.

[18] Sze JY, Victor M, Loer C, Shi Y, Ruvkun G (2000). Food and metabolic signalling defects in a *Caenorhabditis elegans* serotonin-synthesis mutant.. Nature.

[19] McIntire SL, Reimer R, Schuske K, Edwards RH, Jorgensen E (1997). Identification and characterization of the vesicular GABA transporter.. Nature.

[20] Lickteig KM, Duerr JS, Frisby DL, Hall DH, Rand JB (2001). Regulation of Neurotransmitter Vesicles by the Homeodomain Protein UNC-4 and Its Transcriptional Corepressor UNC-37/Groucho in *Caenorhabditis elegans* Cholinergic Motor Neurons.. J Neurosci.

[21] Lee RY, Sawin ER, Chalfie M, Horvitz HR, Avery L (1999). EAT-4, a homolog of a mammalian sodium-dependent inorganic phosphate cotransporter, is necessary for glutamatergic neurotransmission in *Caenorhabditis elegans*.. J Neurosci.

[22] Carvelli L, McDonald PW, Blakely RD, DeFelice LJ (2004). Dopamine transporters depolarize neurons by a channel mechanism.. Proc Natl Acad Sci U S A.

[23] Sulston J, Dew M, Brenner S (1975). Dopaminergic neurons in the nematode *Caenorhabditis elegans*.. J Comp Neur.

[24] Sanyal S, Wintle RF, Kindt KS, Nuttley WM, Arvan R (2004). Dopamine modulates the plasticity of mechanosensory responses in *Caenorhabditis elegans*.. EMBO J.

[25] Stringham EG, Candido EPM (1994). Transgenic hsp16-*lacZ* strains of the soil nematode *Caenorhabditis elegans* as biological monitor of environmental stress.. Environ Tox Chem.

[26] Jones D, Stringham EG, Babich SL, Candido EP (1996). Transgenic strains of the nematode *C. elegans* in biomonitoring and toxicology: effects of captan and related compounds on the stress response.. Toxicology.

[27] Ryu EJ, Harding H P, Angelastro JM, Vitolo OV, Ron D (2002). Endoplasmic reticulum stress and the unfolded protein response in cellular models of Parkinson's disease.. J Neurosci.

[28] Calfon M, Zeng H, Urano F, Till JH, Hubbard SR (2002). IRE1 couples endoplasmic reticulum load to secretory capacity by processing the XBP-1 mRNA.. Nature.

[29] Bush KT, Goldberg AL, Nigam SK (1997). Proteasome inhibition leads to a heat-shock response, induction of endoplasmic reticulum chaperones and thermotolerance.. J Biol Chem.

[30] Bence NF, Sampat RM, Kopito RR (2001). Impairment of the ubiquitin-proteasome system by protein aggregation.. Science.

[31] Gailliot FP, Cannell RJP (1998). Initial extraction and product capture.. Natural Products Isolation.

[32] Ling Z, Gayle DA, Ma SY, Lipton JW, Tong CW (2002). In utero bacterial endotoxin exposure causes loss of tyrosine hydroxylase neurons in the postnatal rat midbrain.. Mov Disord.

[33] Ling ZD, Chang Q, Lipton JW, Tong CW, Landers TM (2004). Combined toxicity of prenatal bacterial endotoxin exposure and postnatal 6-hydroxydopamine in the adult rat midbrain.. Neuroscience.

[34] Pandey UB, Nie Z, Batlevi Y, McCray BA, Ritson GP (2007). HDAC6 rescues neurodegeneration and provides an essential link between autophagy and the UPS.. Nature.

[35] Nedelsky NB, Todd PK, Taylor JP (2008). Autophagy and the ubiquitin-proteasome system: collaborators in neuroprotection.. Biochim Biophys Acta.

[36] Mosharov EV, Larsen KE, Kanter E, Phillips KA, Wilson K (2009). Interplay between cytosolic dopamine, calcium, and alpha-synuclein causes selective death of substantia nigra neurons.. Neuron.

[37] Zeng BY, Bukhatwa S, Hikima A, Rose S, Jenner P (2006). Reproducible nigral cell loss after systemic proteasomal inhibitor administration to rats.. Ann Neurol.

[38] Schapira AH, Cleeter MW, Muddle JR, Workman JM, Cooper RH (2006). Proteasomal inhbition causes loss of nigral tyrosine hydroxlase neurons.. Ann Neurol.

[39] Kordower JH, Kanaan NM, Chu Y, Sursh Babu R, Stansell J (2006). Failure of proteasome inhibitor administration to provide a model of Parkinson's disease in rats and monkeys.. Ann Neurol.

[40] Manning-Bog AB, Reaney SH, Chou VP, Johnston AL, McCormack J (2006). Lack of nigrostriatal pathology in a rat model of proteasome inhibition.. Ann Neurol.

[41] Li X, Du Y, Fan X, Yang D, Luo G (2008). c-Jun N-terminal kinase mediates lactacystin-induced dopamine neuron degeneration.. J Neuropathol Exp Neurol.

[42] Bedford L, Hay D, Devoy A, Paine S, Powe DG (2008). Depletion of 26S proteasomes in mouse brain neurons causes neurodegeneration and Lewy-like inclusions resembling human pale bodies.. J Neurosci.

[43] Kohbata S, Beaman BL (1991). L-Dopa-responsive movement disorder caused by *Nocardia asteroides* localized in the brains of mice.. Infect Immun.

[44] Ogata SA, Beaman BL (1992). Adherence of *Nocardia asteroides* within the murine brain.. Infect Immun.

[45] Ogata SA, Beaman BL (1992). Site-specific growth of *Nocardia asteroides* in the murine brain.. Infect Immun.

[46] Chapman G, Beaman BL, Loeffler DA, Camp DM, Domino EF (2003). In situ hybridization for detection of nocardial 16S rRNA: reactivity within intracellular inclusions in experimentally infected cynomolgus monkeys–and in Lewy body-containing human brain specimens.. Exp Neurol.

[47] Tam S, Barry DP, Beaman L, Beaman BL (2002). Neuroinvasive *Nocardia asteroides* GUH-2 induces apoptosis in the substantia nigra *in vivo* and dopaminergic cells *in vitro*.. Exp Neurol.

[48] Barry DP, Beaman BL (2006). Modulation of eukaryotic cells apoptosis by members of the bacterial order Actinomycetales.. Apoptosis.

[49] Díaz-Corrales FJ, Colasante C, Contreras Q, Puig M, Serrano JA (2004). *Nocardia otitidiscaviarum* (GAM-5) induces parkinsonian-like alterations in mouse.. Braz J Med Biol Res.

[50] Loeffler DA, Camp DM, Qu S, Beaman BL, LeWitt PA (2004). Characterization of dopamine-depleting activity of *Nocardia asteroides* strain GUH-2 culture filtrate on PC12 cells.. Microb Pathog.

[51] Brenner S (1974). The genetics of *Caenorhabditis elegans*.. Genetics.

[52] Maduro M, Pilgrim D (1995). Identification and cloning of *unc-119*, a gene expressed in the *Caenorhabditis elegans* nervous system.. Genetics.

[53] Praitis V, Casey E, Collar D, Austin J (2001). Creation of low-copy integrated transgenic lines in *Caenorhabditis elegans*.. Genetics.

[54] Caldwell GA, Williams SN, Caldwell KA (2006). Integrated Genomics: A Discovery-Based Laboratory Course..

[55] Strange K, Christensen M, Morrison R (2007). Primary culture of *Caenorhabditis elegans* developing embryo cells for electrophysiological, cell biological and molecular studies.. Nature Protocols.

[56] Decker T, Lohmann-Matthes ML (1988). A quick and simple method for the quantitation of lactate dehydrogenase release in measurements of cellular cytotoxicity and tumor necrosis factor (TNF) activity.. J Immunol Methods.

